# The effects of a sugar-sweetened beverage tax and a nutrient profiling tax based on Nutri-Score on consumer food purchases in a virtual supermarket: a randomised controlled trial

**DOI:** 10.1017/S1368980021004547

**Published:** 2022-04

**Authors:** Michelle Eykelenboom, Margreet R Olthof, Maartje M van Stralen, Sanne K Djojosoeparto, Maartje P Poelman, Carlijn BM Kamphuis, Reina E Vellinga, Wilma E Waterlander, Carry M Renders, Ingrid HM Steenhuis

**Affiliations:** 1 Department of Health Sciences, Faculty of Science, Vrije Universiteit Amsterdam, Amsterdam Public Health Research Institute, De Boelelaan 1085, 1081 HV Amsterdam, the Netherlands; 2 Department of Human Geography and Spatial Planning, Faculty of Geosciences, Utrecht University, Utrecht, the Netherlands; 3 Chairgroup Consumption and Healthy Lifestyles, Department of Social Sciences, Wageningen University & Research, Wageningen, the Netherlands; 4 Department of Interdisciplinary Social Science, Faculty of Social and Behavioural Sciences, Utrecht University, Utrecht, the Netherlands; 5 National Institute for Public Health and the Environment (RIVM), Bilthoven, the Netherlands; 6 Department of Public and Occupational Health, Amsterdam UMC, University of Amsterdam, Amsterdam Public Health Research Institute, Amsterdam, the Netherlands

**Keywords:** Food policy, Nutri-score, Randomised controlled trial, Sugar-sweetened beverage tax, Virtual supermarket

## Abstract

**Objective:**

To investigate the effects of a sugar-sweetened beverage (SSB) tax and a nutrient profiling tax on consumer food purchases in a virtual supermarket.

**Design:**

A randomised controlled trial was conducted with a control condition with regular food prices (*n* 152), an SSB tax condition (*n* 130) and a nutrient profiling tax condition based on Nutri-Score (*n* 112). Participants completed a weekly grocery shop for their household. Primary outcome measures were SSB purchases (ordinal variable) and the overall healthiness of the total shopping basket (proportion of total unit food items classified as healthy). The secondary outcome measure was the energy (kcal) content of the total shopping basket. Data were analysed using regression analyses.

**Setting:**

Three-dimensional virtual supermarket.

**Participants:**

Dutch adults aged ≥18 years are being responsible for grocery shopping in their household (*n* 394).

**Results:**

The SSB tax (OR = 1·62, (95 % CI 1·03, 2·54)) and the nutrient profiling tax (OR = 1·88, (95 %CI 1·17, 3·02)) increased the likelihood of being in a lower-level category of SSB purchases. The overall healthiness of the total shopping basket was higher (+2·7 percent point, (95 % CI 0·1, 5·3)), and the energy content was lower (−3301 kcal, (95 % CI −6425, −177)) for participants in the nutrient profiling tax condition than for those in the control condition. The SSB tax did not affect the overall healthiness and energy content of the total shopping basket (*P* > 0·05).

**Conclusions:**

A nutrient profiling tax targeting a wide range of foods and beverages with a low nutritional quality seems to have larger beneficial effects on consumer food purchases than taxation of SSB alone.

Over the past few decades, the prevalence of overweight and obesity has increased worldwide^(1)^. This is alarming as overweight and obesity are associated with an increased risk of several non-communicable diseases including type 2 diabetes, cardiovascular diseases, musculoskeletal disorders and some types of cancer^(1)^. In the European Union, more than half of the adult population are overweight^(2)^. The fundamental cause of overweight and obesity is an imbalance between energy intake and energy expenditure^(1)^. A major driver of a positive energy balance is an unhealthy dietary pattern with excess consumption of energy-dense, ultra-processed foods containing high levels of added sugar and saturated fat^(1)^. The food environment plays a crucial role in food choice and a shift towards healthier dietary patterns requires interventions within the physical, sociocultural, political and economic food environment^(3,4)^.

In the economic food environment, food price is an important determinant of food choice^(5)^. Fiscal policies form a promising strategy to stimulate healthy food choices^(6)^. A policy that has received considerable attention in recent years is taxation of sugar-sweetened beverages (SSB). In 2016, the WHO recommended governments to tax SSB^(7)^. The rationale for targeting SSB includes compelling evidence that the consumption of SSB is causally associated with weight gain and that SSB provide no nutritional value^(8–10)^. There is substantial evidence that taxation of SSB reduces purchases and consumption of SSB and stimulates the beverage industry to reformulate their products to reduce sugar content^(11–13)^. Over forty countries worldwide have implemented an SSB tax in various forms^(14)^. The World Cancer Research Fund International provides an overview of implemented SSB taxes^(14)^. For example, a two-tiered specific excise tax implemented in 2018 in the UK, the Soft Drink Industry Levy (SDIL), applies different tax rates depending on the sugar content of beverages^(14)^. Health-related food taxes targeting a wide range of unhealthy foods and beverages are only implemented in Mexico and Hungary^(14)^. In Mexico, non-essential foods with an energy density of ≥275 kcal/100 g are taxed^(14)^. The Hungarian ‘Public Health Product Tax’ targets products high in sugar, salt and/or caffeine^(14)^.

Although the focus of most food tax initiatives has been on SSB, taxes targeting a wider range of unhealthy foods and beverages may have more beneficial effects on healthy food choice than taxation of SSB alone^(15,16)^. Waterlander *et al.* measured the effects of food price variations simulating different policies, including a beverage tax and three different nutrient taxes, on consumer food purchasing in a New Zealand virtual supermarket setting^(17)^. The beverage tax did not affect study outcomes, while the three nutrient taxes – a saturated fat tax, sugar tax and salt tax – all had independent, positive effects on healthy food purchases and their target nutrient. However, the saturated fat tax and salt tax also led to an increase in the purchases of sugar in products as a percentage of total purchased energy. A solution to such potential unintended substitution effects might be to target several nutrients and/or foods with one tax simultaneously, using a nutrient profiling model^(6,18)^. Poelman *et al.* measured the effects of a nutrient profiling tax based on the WXY^(19)^ nutrient profiling model in a Dutch virtual supermarket^(20)^. Poelman *et al.* did not find significant effects on the purchases of kcal, sugar and saturated fat, likely as a consequence of low statistical power^(20)^.

In Europe, the nutrient profiling model Nutri-Score has been adopted for front-of-pack labelling purposes in France, Belgium, Spain, Germany, Switzerland and Luxembourg and is currently being discussed in many other countries, such as in the Netherlands^(21)^. Nutri-Score is based on the British Food Standards Agency nutrient profiling system and presents the nutritional quality of food and beverage products on a five-point, colour-coded scale^(22)^. In addition to front-of-pack labelling purposes, Nutri-Score could also be helpful in determining which foods and beverages should be subject to health-related food taxes^(6)^. To the best of our knowledge, there have not been randomised controlled intervention studies investigating the effects of taxation of SSB alone and taxation of a wider range of unhealthy foods and beverages by the use of the Nutri-Score on consumer food purchases. As part of the European Policy Evaluation Network^(23)^, the aim of this study was therefore to investigate the effects of an SSB tax based on a scheme similar to an enacted European SSB tax – the UK’s SDIL – and a nutrient profiling tax based on Nutri-Score on SSB purchases and healthy food purchases in a virtual supermarket setting.

## Methods

### Study design

A randomised controlled trial was conducted in which participants were randomly assigned to one of the following conditions in the virtual supermarket: (i) a control condition with regular food prices; (ii) an experimental condition with a two-tiered SSB tax or (iii) an experimental condition with a nutrient profiling tax based on Nutri-Score^(22)^. In the Netherlands, regular food prices include a value-added tax rate of 9 % that applies to all food and beverage products^(24)^. Moreover, a consumption tax of €0·0883/l applies to fruit and vegetable juices, soft drinks and mineral water, with no distinction between SSB and sugar-free beverages (e.g. water or non-caloric sweetened beverages)^(25)^. To reflect a real-world situation in which people would likely be aware of the implementation of food taxes^(26,27)^, participants in the experimental conditions were informed about the tax before entering the virtual supermarket with a notification. The notification was tailored to the condition; ‘In the virtual supermarket, beverages high in sugar are taxed’ or ‘In the virtual supermarket, unhealthy products high in sugar, fat and/or salt (such as biscuits, sweets, snacks and soft drinks) are taxed’. Participants in the control condition did not receive such a notification.

In the experimental condition with SSB taxation, beverages were taxed on a scheme similar to the UK’s SDIL. This meant that beverages containing 5–8 g of sugar/100 ml were additionally taxed €0·21/l and beverages containing 8 g of sugar or more per 100 ml were additionally taxed €0·28/l^(14)^. In line with the SDIL, the tax was not applied to milk-based drinks, milk substitute drinks, alcohol substitute drinks and 100 % fruit juices without added sugar^(14)^. We selected the SDIL because it is regarded as an effective SSB tax in Europe with its tiered tax system and relatively high tax rate^(14)^. The implementation of the SDIL accelerated the decline in the sale of sugar from soft drinks in the UK as a result of changes in consumer purchasing and action by the SSB industry to reduce sugar in products^(28)^. In the virtual supermarket, the SSB tax corresponded to an average price increase of 22 % for the beverages liable for the tax. In total, 34 SSB (6 % of the stock of the virtual supermarket and 40 % of the non-alcoholic beverages) were taxed (see Table 1). The prices of other products were identical to the control condition.

**Table 1:** Overview of food categories and the number of taxed products in the experimental conditions in the virtual supermarket

Food category*	Total products (*n*)	Taxed products in the SSB tax condition (*n*)	%	Taxed products in the nutrient profiling tax condition (*n*)	%
Potatoes and tubers	11	–		–	
Alcoholic beverages	17	–		–	
Bread	26	–		4	
Miscellaneous foods	2	–		–	
Eggs	2	–		–	
Fruits	18	–		–	
Pastry and biscuits	31	–		22	
Cereals and cereal products	26	–		3	
Vegetables	42	–		1	
Savoury bread spreads	9	–		4	
Savoury sauces	22	–		8	
Savoury snacks	22	–		13	
Cheese	20	–		19	
Herbs and spices	27	–		16	
Milk and milk products	51	–		15	
Non-alcoholic beverages†	86	34		34	
Nuts and seeds	9	–		2	
Legumes	4	–		–	
Mixed dishes	23	–		–	
Soups	9	–		–	
Sugar, sweets and sweet sauces	48	–		41	
Fats and oils	10	–		8	
Fish	10	–		1	
Meat and poultry	25	–		11	
Meat substitutes and dairy substitutes	11	–		4	
Cold meat cuts	19	–		18	
Total	580	34	6	224	39

* Classification from the Dutch food composition database (NEVO)^(33)^.

† Detailed information can be found in Supplemental Table S1.

In the nutrient profiling tax condition, taxation of energy-dense, nutrient-poor foods and beverages was based on the nutrient profiling scheme Nutri-Score^(22)^. The five-point, colour-coded scale of Nutri-Score ranges from most healthy (dark green, associated with the letter ‘A’) to least healthy (red, associated with the letter ‘E’)^(21)^. In this study, food and beverage products with the label ‘D’ or ‘E’ were classified as unhealthy. The prices of these products were increased by 20 % based on previous scientific evidence suggesting that such price increases would be needed to have meaningful effects on purchases, consumption and ultimately population health^(6,12)^. In total, 224 food and beverage products (39 % of the stock of the virtual supermarket) were taxed (see Table 1). Similar to the SSB tax condition, 34 SSB were taxed in the nutrient profiling tax condition. The prices of other products were identical to the control condition.

### Setting: the virtual supermarket

The present study was conducted in a Dutch virtual supermarket^(29)^, which is a three-dimensional software application that simulates the in-store environment of a real supermarket. A validation study showed that food purchasing behaviour in the virtual supermarket is comparable to real-life food purchasing behaviour^(30)^. Shopping in the virtual supermarket closely mirrors a real-life supermarket experience; participants can move along supermarket aisles and place products in their basket with a single mouse click. More detailed information on the main features of the software application can be found elsewhere^(29)^.

The original version of the Dutch virtual supermarket^(29)^ has been updated between January 2019 and March 2020 for the purpose of this study. The software update included several new features to create a more realistic simulation of a real-life supermarket experience (e.g. in-store signage, cash register shelves and ambient supermarket sound effects) and to facilitate the use of the software (e.g. a tutorial prior to the experiment). In addition, twenty products (e.g. meat substitutes and freshly baked bakery products) were added to the stock of the virtual supermarket to align with the changed stock over the past years. The identification of new products was conducted by the research team and based on the stock of the leading supermarket chain in the Netherlands and the most frequently consumed foods according to the most recent Dutch National Food Consumption Survey (2012–2016)^(31)^. Since we did not have access to sales data, the number and type of products within food and beverage categories as shown on the website of the leading supermarket chain were used to model the usual stock. Moreover, for the specific purpose of this trial, forty-eight non-alcoholic beverages (of which twenty-six SSB) were added. We included different package sizes and selected the supermarket’s own-brand and the most common premium brand for the beverages, which enabled us to investigate potential shifts from more expensive premium brand beverages to cheaper supermarket’s own-brand beverages as a consequence of the taxes in the experimental conditions^(32)^. No distinction was made between different brands for other food categories. The updated virtual supermarket contained 580 food products, including 119 beverages. Food prices were updated using the website of the leading supermarket chain in February 2020. Each product was priced with the average price of the supermarket’s own brand and the most common premium brand of that product weighted for pack size, with the exception of the non-alcoholic beverages which were priced depending on the brand. Moreover, information on the nutritional composition of the products was updated using the online Dutch Food Composition Database (NEVO) version 2019^(33)^. Nutri-Scores were calculated using a calculation tool of the French National Public Health Agency^(22)^. The updated virtual supermarket was pilot-tested on a heterogeneous population of Dutch adults (*n* 13) who were asked for feedback on the download instructions and software manuals, user-friendliness of the software as well as the closing questionnaire. Based on the feedback, we improved the lay-out of the instructions, the display settings for the visibility of products on bottom shelves and the phrasing of questions in the closing questionnaire. All study materials were developed in Dutch language proficiency level B1, which is understood by the vast majority of the population^(34)^.

### Participants and recruitment

Participants were eligible for inclusion in the study if they met the following criteria: (i) being 18 years or older; (ii) being familiar with the Dutch language; (iii) being largely/totally responsible for grocery shopping in their household and (iv) having access to a laptop or computer. Participants were recruited between June and August 2020 using one of the largest online research panels in the Netherlands with more than 100 000 members (Panel Inzicht)^(35)^. Participation was rewarded with panel member points that could be redeemed for cash (€4·00). We aimed to recruit a sample with an equal distribution of participants with a low (elementary, lower secondary or lower vocational), moderate (higher secondary or intermediate vocational) and high (higher vocational or university) educational level by applying quotas. The classification of educational level was based on the standard classification from Statistics Netherlands (CBS)^(36)^. An a priori sample size estimation indicated that 327 participants (109 per intervention group) would be required to detect a difference of 0·8 l SSB purchases/household per week with 80 % power at a two-sided 5 % level of significance^(37,38)^. The recruitment was continued until the required number of 109 participants was achieved for all research conditions. This approach resulted in some of the research conditions in a number of participants that was higher than required. Due to lower than expected recruitment in the first 2 months, particularly among those with a low educational level, additional efforts were undertaken to promote the recruitment in August (e.g. by means of additional reminders and an instruction video).

The trial protocol was registered at the Netherlands Trial Register (registration number NL8616). In parallel with the recruitment for this study, we recruited participants for experimental conditions for another project which aims to evaluate the effects of a meat tax, an information nudge and a combination of both on consumer meat purchases (Netherlands Trial Register registration number NL8628). The data from participants in the control condition were used as a reference to measure the effects of all experimental conditions. As the participants in the control condition conducted a grocery shopping task without being exposed to an intervention and without being aware of the purpose of the study, the use of data from participants in the control condition as a reference for two different research questions could not have affected their purchasing behaviour in the virtual supermarket.

### Procedures

The entire study was executed online. The eligibility screening, informed consent and randomisation procedure were conducted by the research panel, who had no involvement in the study design, analysis of the data or interpretation of the results. Random subsamples of panel members were sent an email invitation to participate in the study. Following eligibility screening and informed consent, participants received a download link and a log-in code to enter the virtual supermarket. Participants were randomised using a computer-generated list of log-in codes. The log-in codes corresponded with random allocation to one of the research conditions. Before entering the virtual supermarket, participants were asked about their household size and household composition (i.e. the number of people in the age categories 0–4 years, 4–9 years, 9–14 years and 14 years or older) to allocate a household-specific weekly grocery shopping budget according to data provided by the National Institute for Family Finance Information^(39)^. For a two-adult-household, this budget was 89 euros, whereas for a household with two adults and two children in the age of 9–14 years, the budget was 117 euros. Participants were instructed to conduct a typical weekly grocery shop for their household in the virtual supermarket. Moreover, participants in the experimental conditions received the tax notification. To help participants to become familiar with the software, participants had to complete a tutorial prior to the experiment in which they had to find three pre-determined products in the virtual supermarket. Next, participants were able to conduct their weekly grocery shop for their household. When finished shopping, participants moved to the cash register and completed a closing questionnaire. Participants did not actually receive the purchases they made.

### Measures

Primary outcome measures were SSB purchases in l/household per week and overall healthiness of the total weekly food shopping basket (proportion of total unit food items with a Nutri-Score label ‘A’, ‘B’ or ‘C’). The secondary outcome measure was the energy (kcal) content of the total weekly food shopping basket. Information on the number of total unit food items purchased, own-brand SSB purchases as a proportion of total SSB purchases and SSB purchases as a proportion of total non-alcoholic beverage purchases was also collected.

In the closing questionnaire, participants reported on demographic characteristics (see Table 2). Moreover, several questions on factors that may influence shopping behaviour were included. Participants were asked about their understanding of the virtual supermarket and the comparability of their purchases in the virtual supermarket with real-life purchases on a five-point Likert scale ranging from 1 ‘strongly disagree’ to 5 ‘strongly agree’. Acceptability of health-related food taxes was assessed using the items ‘I support imposing an SSB tax in the Netherlands (i.e. a tax on regular soft drinks, fruit juices with added sugar, sports drinks, energy drinks and flavoured water with added sugar)’ and ‘I support imposing a tax on unhealthy foods in the Netherlands (e.g. on products high in sugar, fat and/or salt, such as biscuits, sweets, snacks and soft drinks)’ indicated on a five-point Likert scale ranging from 1 ‘strongly disagree’ to 5 ‘strongly agree’. The acceptability variables were grouped into three categories: ‘Disagree’ (response options 1 and 2), ‘Neither’ (response option 3) and ‘Agree’ (response options 4 and 5). In an open-ended question, participants were asked whether they purchased different foods and/or beverages due to COVID-19. Answers were categorised into ‘Yes’ and ‘No’ by the research team. Price awareness was assessed using the items ‘To what extent did you notice prices in the virtual supermarket’ and ‘To what extent did prices influence your choices in the virtual supermarket’ indicated on a seven-point Likert scale ranging from 1 ‘not at all’ to 7 ‘extremely’. Finally, participants in the experimental conditions were asked whether they noticed the tax notification and to what extent this notification influenced their choices in the virtual supermarket on a seven-point Likert scale ranging from 1 ‘not at all’ to 7 ‘extremely’.

**Table 2:** Descriptive statistics of the study participants

	Total (*n* 394)		Control condition (*n* 152)		SSB tax condition (*n* 130)		Nutrient profiling tax condition (*n* 112)	
	*n* or mean	% or sd	*n* or mean	% or sd	*n* or mean	% or sd	*n* or mean	% or sd
Age (years), mean and sd	48·5	15·7	48·6	16·3	48·6	15·1	48·3	15·6
Sex, *n* and %								
Female	216	54·8	78	51·3	81	62·3	57	50·9
Male	178	45·2	74	48·7	49	37·7	55	49·1
Educational level, *n* and %								
Low	66	16·8	20	13·2	21	16·2	25	22·3
Moderate	144	36·5	44	28·9	57	43·8	43	38·4
High	184	46·7	88	57·9	52	40·0	44	39·3
BMI (kg/m^2^)*, mean and sd	26·7	5·8	27·5	6·0	26·5	5·7	26·0	5·4
Weight status*, *n* and %								
BMI < 25 kg/m^2^	178	46·1	65	43·0	61	48·4	52	47·7
Overweight	128	33·2	49	32·5	38	30·2	41	37·6
Obese	80	20·7	37	24·5	27	21·4	16	14·7
Household size, mean and sd	2·3	1·2	2·3	1·2	2·4	1·3	2·4	1·2
Household composition, mean and sd								
% of household 14 years or older	91·8	17·8	91·4	18·6	91·3	18·1	92·9	16·4
Employment status, *n* and %								
Employed	230	58·4	85	55·9	79	60·8	66	58·9
Retired	64	16·2	30	19·7	17	13·1	17	15·2
Other	100	25·4	37	24·3	34	26·2	29	25·9
Household monthly income (gross in €)†, *n* and %								
Low (0–2000)	107	27·2	38	25·0	36	27·7	33	29·5
Medium (2000–3000)	97	24·6	37	24·3	31	23·8	29	25·9
High (3000+)	190	48·2	77	50·7	63	48·5	50	44·6
Household weekly food expenditures (in €), *n* and %								
0–60	131	33·2	52	34·2	38	29·2	41	36·6
60–100	139	35·3	54	35·5	47	36·2	38	33·9
100+	124	31·5	46	30·3	45	34·6	33	29·5
Shopping budget in virtual supermarket (€), mean and sd	88·1	34·5	86·5	33·7	86·7	28·6	91·7	41·3
% of budget spent, mean and sd	82·1	21·1	84·1	19·8	82·2	21·3	79·3	22·4
Total expenditure (€), mean and sd	71·5	30·5	71·6	29·3	71·4	31·0	71·5	31·8
Appreciation of shopping budget, *n* and %								
More than usual	131	33·2	49	32·2	35	26·9	47	42·0
Same as usual	183	46·4	70	46·1	63	48·5	50	44·6
Less than usual	80	20·3	33	21·7	32	24·6	15	13·4
Understanding virtual supermarket‡, mean and sd	4·5	0·6	4·5	0·6	4·5	0·7	4·6	0·6
Comparability to real-life purchases§, mean and sd	4·0	0·8	4·0	0·8	4·0	0·8	4·1	0·8
Changed purchases due to COVID-19, *n* and %								
Yes	71	18·0	29	19·1	21	16·2	21	18·8
No	323	82·0	123	80·9	109	83·8	91	81·3
Price awareness‖, mean and sd	3·9	1·6	3·9	1·6	3·8	1·7	3·8	1·5
Awareness of taxation, *n* and %								
Yes	NA		NA		101	77·7	85	75·9
No	NA		NA		29	22·3	27	24·1
Influence of notification¶, mean and sd	NA		NA		1·8	1·3	2·3	1·5
Acceptability of an SSB tax**, *n* and %								
Disagree	135	34·3	45	29·6	46	35·4	44	39·3
Neutral	82	20·8	31	20·4	32	24·6	19	17·0
Agree	177	44·9	76	50·0	52	40·0	49	43·8
Acceptability of an unhealthy food and beverages tax††, *n* and %								
Disagree	141	35·8	45	29·6	50	38·5	46	41·1
Neutral	82	20·8	33	21·7	27	20·8	22	19·6
Agree	171	43·4	74	48·7	53	40·8	44	39·3

* *n* 386.

† The median gross monthly income in the Netherlands (2018) is €4450^(56)^.

‡ Measured by one item ‘The program was easy to understand’ indicated on a five-point Likert scale ranging from 1 ‘strongly disagree’ to 5 ‘strongly agree’.

§ Measured by one item ‘The products I have purchased in the virtual supermarket are comparable to my regular food purchases in real-life’ indicated on a five-point Likert scale ranging from 1 ‘strongly agree’ to 5 ‘strongly agree’.

‖ Measured by two items ‘To what extent did you notice prices in the virtual supermarket?’ and ‘To what extent did prices influence your choices in the virtual supermarket?’ indicated on a seven-point Likert scale ranging from 1 ‘not at all’ to 7 ‘extremely’.

¶ Measured by one item ‘To what extent did the notification influence your choices in the virtual supermarket?’ indicated on a seven-point Likert scale ranging from 1 ‘not at all’ to 7 ‘extremely’.

** Measured by one item ‘I support imposing an SSB tax in the Netherlands (e.g. on regular soft drinks, fruit juices with added sugar, sports drinks, energy drinks and flavoured water with added sugar)’ indicated on a five point-Likert scale ranging from 1 ‘strongly disagree’ to 5 ‘strongly agree’.

†† Measured by one item ‘I support imposing a tax on unhealthy foods in the Netherlands (e.g. on products high in sugar, fat and/or salt, such as biscuits, sweets, snacks and soft drinks)’ indicated on a five point-Likert scale ranging from 1 ‘strongly disagree’ to 5 ‘strongly agree’.

### Statistical analyses

Descriptive statistics were used to characterise the sample. Outcome measures were assessed for an adequate normal distribution. Due to the highly skewed distribution of SSB purchases, this outcome was transformed into an ordinal variable. As a large proportion of the participants did not purchase any SSB in the virtual supermarket, ‘0 l’ was the first category of this ordinal variable. Eyeballing of the data revealed that SSB purchases followed an exponential distribution. Therefore, the ranges of the subsequent categories were doubled in size to fit the data and to optimise the distribution of participants across the different categories: ‘0·75–1·5 l’, ‘1·5–3 l’, ‘3–6 l’ and ‘6 l or more’. None of the participants purchased between 0 and 0·75 l of SSB. The ordinal variable was analysed using an ordinal regression analysis, which enabled us to investigate whether being in the experimental conditions increased the likelihood of having a lower level of SSB purchases as compared to the control condition. The overall healthiness and energy content of the total weekly food shopping basket followed a normal distribution and were analysed using linear regression analyses.

Effect modification by educational level was tested for the primary outcomes as the literature suggests that lower socioeconomic groups may be more responsive to food taxes and more likely reduce their purchases of taxed products as a result^(6)^. Effect modification was tested by including educational level and interaction terms between the research conditions and educational level in the unadjusted regression models. If an interaction term was statistically significant, stratified analyses were planned for educational level. If no effect modification was present, it was planned to add educational level as a covariate to the analyses. Covariates were added to the unadjusted regression models. Two models were made; model 1 was adjusted for household size as it was proven to be a strong predictor of the outcomes, and model 2 was additionally adjusted for sex, educational level and BMI to correct for imbalances in randomisation. Participants with extreme outliers (more than 3 * IQR below Q1 or above Q3) in any of the outcomes were excluded from all analyses. Moreover, participants who purchased less than or equal to five different products in the virtual supermarket were excluded from the analyses as this was considered implausible for a weekly grocery shop. Statistical analyses were performed using the software IBM SPSS Statistics 26.0. All statistical tests were two-sided and values of *P* < 0·05 were considered statistically significant.

## Results

**Fig. 1:**
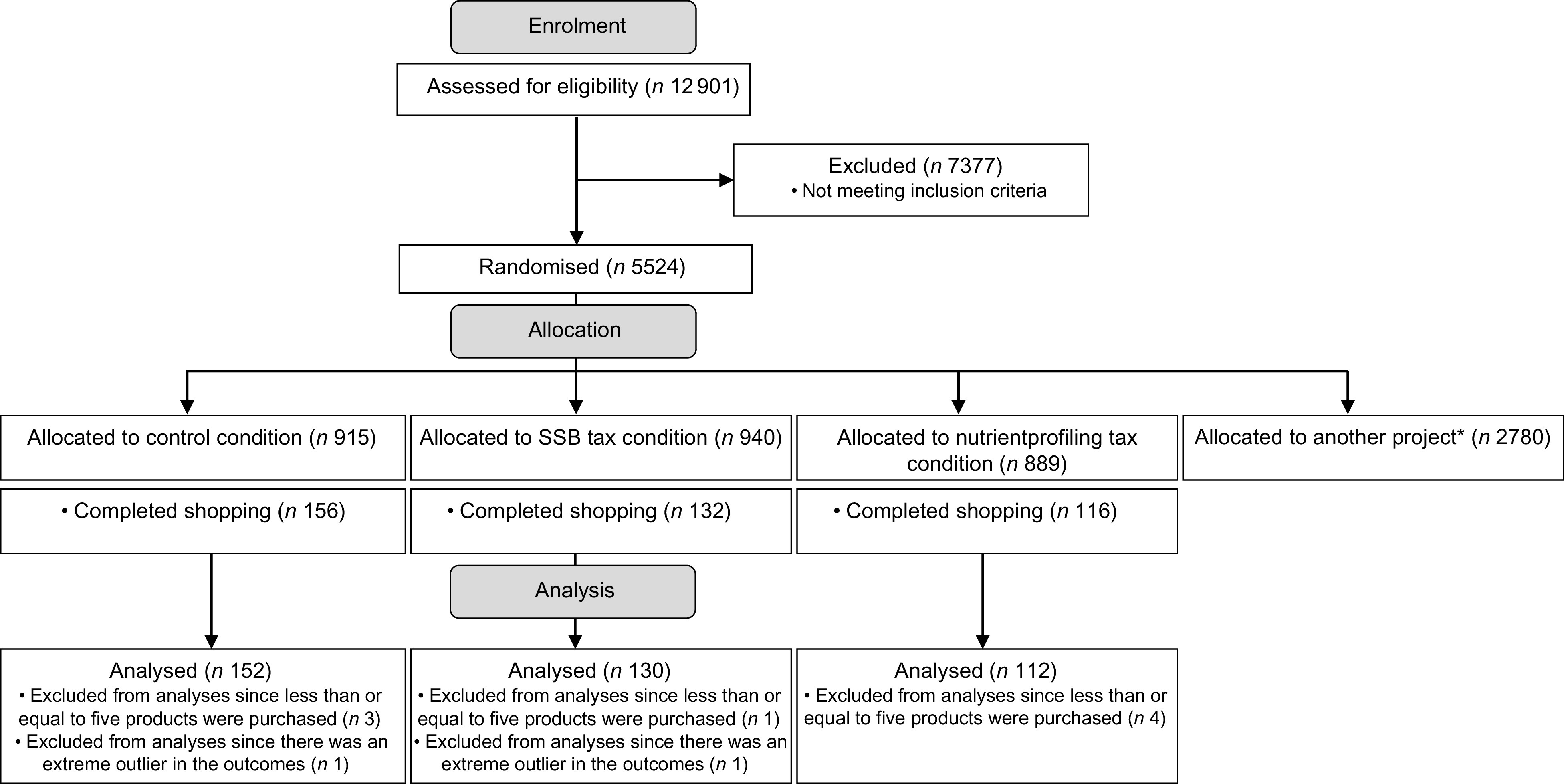
Flowchart of enrolment and allocation of the study participants. *2780 participants were randomised for the purpose of another project (Netherlands Trial Register registration number NL8628)

Between June and August 2020, 150 514 panel members were invited to participate of whom 12 901 participants (8·6 %) completed the screening questionnaire (Fig. 1). A total of 5524 participants (42·8 % of those who completed the screening questionnaire) were eligible for inclusion of whom 2744 participants were randomly assigned to one of the research conditions of this study and 2780 participants to one of the research conditions of another project (Netherlands Trial Register registration number NL8628). Overall, 404 participants (14·7 % of those randomly assigned to this study) completed their shop in the virtual supermarket. The mean age of participants that completed their shop was lower than that of participants that did not complete their shop (see online Supplemental Table S2). Moreover, they more often had a high educational level. The final sample included 394 participants. The characteristics of these participants are presented in Table 2.

### Effect modification

None of the interactions terms was statistically significant (*P* > 0·05). The results were therefore not stratified by educational level.

### SSB purchases

Descriptive statistics of the consumer food purchases in the virtual supermarket are presented in Table 3. In the fully adjusted models, the likelihood of being in a lower-level category of SSB purchases was 1·62 times higher (95 % CI 1·03, 2·54) in the SSB tax condition compared with the control condition (Table 4). In the nutrient profiling tax condition, the likelihood of being in a lower-level category of SSB purchases was 1·88 times higher (95 % CI 1·17, 3·02) compared with the control condition (Table 4).

**Table 3:** Descriptive statistics of the consumer food purchases in the virtual supermarket

	Total		Control condition		SSB tax condition		Nutrient profiling tax condition	
	*n* or mean or median	% or sd or IQR	*n* or mean or median	% or sd or IQR	*n* or mean or median	% or sd or IQR	*n* or mean or median	% or sd or IQR
SSB (l), median and IQR	0·8	3·0	1·5	3·0	0·8	3·0	0·8	2·2
SSB, *n* and %								
0 l	176	44·7	58	38·2	63	48·5	55	49·1
0·75–1·5 l	30	7·6	10	6·6	10	7·7	10	8·9
1·5–3 l	84	21·3	39	25·7	23	17·7	22	19·6
3–6 l	75	19·0	31	20·4	26	20·0	18	16·1
6 l or more	29	7·4	14	9·2	8	6·2	7	6·3
Own-brand SSB (% of total SSB)*, median and IQR	66·7	100·0	73·2	100·0	60·2	100·0	55·6	100·0
SSB (% of total non-alcoholic beverages)†, median and IQR	37·0	74·3	49·5	73·2	21·8	77·6	29·2	63·3
Total unit food items (*n*), mean and sd	39·7	17·6	40·1	16·8	40·5	18·6	38·2	17·6
Proportion healthy (%), mean and sd	71·6	10·9	70·8	10·2	71·8	11·0	72·5	11·5
Total energy (kcal), mean and sd	32 212	17 038	32 447	16 488	33 338	18 686	30 586	15 745

* 218.

† 347.

**Table 4:** Effects of the experimental conditions on the likelihood of being in a lower-level category of SSB purchases using ordinal regression analyses

	SSB tax condition			Nutrient profiling tax condition		
	OR§	95 % CI	*P*	OR§	95 % CI	*P*
Model 1†	1·56	1·01, 2·41	0·046*	1·71	1·08, 2·72	0·022*
Model 2‡	1·62	1·03, 2·54	0·037*	1·88	1·17, 3·02	0·010*

* *P* < 0·05.

† Adjusted for household size.

‡ Adjusted for household size, sex, educational level and BMI.

§ Compared with the control condition.

### Overall healthiness and energy content of the total weekly food shopping basket

In the fully adjusted models, the proportion of total unit food items classified as healthy was on average 2·7 percent point (95 % CI 0·1, 5·3) higher for participants in the nutrient profiling tax condition than for those in the control condition (Table 5). The total amount of kcal purchased was on average 3301 kcal (95 % CI −6425, −177) lower for participants in the nutrient profiling tax condition than for those in the control condition (Table 5). The SSB tax did not affect the overall healthiness and energy content of the total weekly food shopping basket (Table 5).

**Table 5:** Effects of the experimental conditions on the overall healthiness and energy content of the total weekly food shopping basket using linear regression analyses

	SSB tax condition			Nutrient profiling tax condition		
	*B* §	95 % CI	*P*	*B* §	95 % CI	*P*
Proportion healthy (%)						
Model 1†	1·2	−1·4, 3·7	0·369	1·8	–0·8, 4·4	0·183
Model 2‡	1·4	−1·2, 3·9	0·293	2·7	0·1, 5·3	0·044*
Total energy (kcal)						
Model 1†	−425	−3361, 2511	0·776	−2627	–5685, 432	0·092
Model 2‡	−1452	−4447, 1543	0·341	−3301	–6425, −177	0·038*

* *P* < 0·05

† Adjusted for household size.

‡ Adjusted for household size, sex, educational level and BMI.

§ Compared with the control condition.

## Discussion

This study aimed to investigate the effects of an SSB tax and a nutrient profiling tax based on Nutri-Score on SSB purchases and healthy food purchases in a virtual supermarket. We demonstrated that the nutrient profiling tax is effective in decreasing SSB purchases as well as in increasing the overall healthiness and decreasing the energy content of the total weekly food shopping basket; in case of an SSB tax, effects were only observed on SSB purchases.

The observed reduction in SSB purchases as a result of the SSB tax is in line with previously conducted experimental, modelling and real-world evaluation studies^(11–13)^. Although we statistically did not observe significant effects of the SSB tax on the overall healthiness and energy content of the total weekly food shopping basket, the outcomes were in the expected direction (i.e. a higher proportion of total unit food items classified as healthy and a lower amount of kcal purchased). This finding is consistent with the results of a randomised controlled trial in a New Zealand virtual supermarket setting reported by Waterlander *et al.* ^(17)^. A likely explanation for the lack of significant effects of the SSB tax on the overall healthiness and energy content of the total weekly food shopping basket is that SSB purchases account for only a small part of total food purchases^(17)^, resulting in limited statistical power. Also, SSB might have been substituted with other less healthy and/or high-calorie products. Research on substitution and complementary effects of an SSB tax has been inconclusive. While substitution to other high-calorie beverages such as milk^(16,40)^ and fruit juices^(41)^ was found in some studies, others reported no substitution to other beverages^(42)^ or substitution to low-calorie beverages such as diet soft drinks^(43)^, coffee and tea^(37,44)^. There is no evidence for substitution to sugary foods such as sweets, candies, cookies and desserts^(16,37,41,42)^.

The nutrient profiling tax decreased SSB purchases while it also beneficially affected the overall healthiness and energy content of the total weekly food shopping basket. Compared with the control condition, the nutrient profiling tax decreased the energy content of the total weekly food shopping basket with 3301 kcal/household per week, which would translate into a difference of 205 kcal/person per d based on a mean household size of 2·3 persons. As the Dutch National Food Consumption Survey 2012–2016 showed that Dutch people consume on average 2192 kcal/d^(45)^, this would implicate a substantial reduction in kcal purchased. Our findings are consistent with a modelling study utilising Chilean expenditure data by Caro *et al.* ^(16)^. Caro *et al.* showed that a 30 % tax on all unhealthy foods and beverages (i.e. exceeding thresholds on added sugar, saturated fat and sodium and for which marketing is restricted based on a Chilean law) seems more effective in reducing purchases of calories than an SSB tax^(16)^. A systematic review by Thow *et al.* showed that seven of the eight studies on nutrient profiling taxes found reductions in target food consumption^(13)^. Nutrient profiling taxes targeting several nutrients and/or foods with one tax simultaneously might yield the best results in improving diets as those taxes are not likely to have unintended substitution effects^(13,17)^.

Although this study provides insight into the effects of an SSB tax and a nutrient profiling tax based on Nutri-Score on consumer food purchases, it is important to consider that the effects of the price changes in the virtual supermarket are not identical to the effects that one might expect from health-related food taxes in the real world. Health-related food taxes would likely have a ‘signalling effect’ in the real world, which means that the publicity surrounding the implementation of the taxes may raise public awareness about the negative health consequences of the consumption of the taxed products^(26,46)^. Although a majority of the participants in the experimental conditions reported being aware of the taxes in the virtual supermarket (78 % in the SSB tax and 76 % in the nutrient profiling tax condition), the rationale for implementing the taxes was not communicated. This may imply that the implementation of an SSB tax and a nutrient profiling tax in a real-world setting may have a larger impact on food purchases than in our virtual supermarket setting. Also, reformulation of products by the food and beverage industry can be expected from health-related food taxes in real-world settings, particularly from taxes with tiered tax designs^(6,15,28)^. This study does not account for such changes in the supply side. Moreover, taxes may not change retail prices uniformly in the real world. Evidence indicates that the pass-through of an SSB tax, that is, the percentage of the tax that is passed on to consumers in the form of higher prices, varies by location^(47)^. This is for example illustrated by a review of the effects of SSB taxes on prices that showed that pass-through rates ranged from less than 50 % in Berkeley to 100 % in Philadelphia^(47)^. In the virtual supermarket, we assumed a pass-through rate of 100 %. For situations where taxes are not fully passed on to consumers or for situations where taxes are spread to untaxed products^(48)^, our results might overestimate the real-world effects.

In addition to the potential effectiveness of health-related food taxes on consumer food purchases in the real world, the feasibility of their adoption and implementation in practice needs to be considered as well. Taxation of SSB is regarded as the most feasible health-related food tax to implement^(6)^. Several European countries have successfully implemented an SSB tax, mostly levied as specific excise taxes based on the sugar content of the beverages^(14)^. Taxes targeting a wider range of unhealthy foods and beverages are more complex than SSB taxes^(15)^. Currently, there is no legislative precedent for a tax defined by a nutrient profiling model alone^(14,49)^. There are examples of successful implementation of health-related food taxes using a combination of nutrition criteria and food categories, for example, in Hungary and Mexico^(14,49)^. In our study, the prices of unhealthy foods and beverages were increased by 20 % in the nutrient profiling tax^(14)^. Such price changes could be achieved in the real world by changes to existing value-added taxes, which are calculated as a percentage of the retail price. However, the use of differential value-added taxes on foods can lead to distortions and adds administrative complexity, and is therefore discouraged in global recommendations for tax reform^(50)^. The use of specific excise taxes (i.e. taxes levied based on quantity) seems to be more appropriate and feasible^(50)^. Another factor affecting the feasibility of the adoption and implementation of health-related food taxes is the extent to which they are likely to be acceptable to the public^(51)^. An SSB tax and a tax on unhealthy foods were supported by 45 % and 43 % of the participants in our study, respectively. It is, however, important to note that our sample may not be representative of all adults in the Netherlands and that these percentages may therefore not be generalisable to the Dutch adult population. In an online survey among adults representative of the Dutch population for age, sex, educational level and location, 40 % of the participants supported an SSB tax^(52)^. Public acceptability of an SSB tax in the Netherlands tends to be higher (55 %) if revenue is used for health initiatives^(52)^.

The main strength of this study is that it investigated the effects of taxation of SSB alone and taxation of a wider range of unhealthy foods and beverages by the use of the Nutri-Score in the same controlled setting. A previous validation study showed that food purchasing behaviour in the virtual supermarket is comparable to real-life food purchasing behaviour^(30)^, and a majority of the participants in our study (80 %) indicated that their purchases in the virtual supermarket were comparable to their purchases in real life. In addition, our sample was relatively large compared with other virtual supermarket studies. Also, much effort was made to include participants with a low or moderate educational level to improve the external validity of our findings for the Dutch population. In our sample, 17 % of the participants had a low educational level and 37 % a moderate educational level, which is more or less comparable with the distribution of educational level within the Dutch adult population (23 % and 40 %, respectively)^(53)^.

This study also has several limitations. Although the virtual supermarket has previously been validated against real shopping data^(30)^, an important limitation of this study is that the virtual supermarket is not identical to a real supermarket. Participants did not spend their own money nor did receive the purchases they made, which may have caused them to put less emphasis on prices than in a real supermarket setting involving real money. This may have resulted in an underestimation of the real-world effects. Also, it is important to consider that the product range of the virtual supermarket is less extensive than a real supermarket. The virtual supermarket may therefore not fully take into account complex phenomena such as substitution and complementary effects within food categories^(29)^. Another limitation of this study is that the external validity for the Dutch population may be lower than expected. A large number of participants dropped out after randomisation, although major efforts have been made to minimise drop out. For example, the virtual supermarket was extensively tested, study materials were developed in Dutch language proficiency level B1 and several reminders were sent. We do not have insights into the reasons for drop out, but an explanation for the higher drop-out rates among older participants and participants with a lower educational level might be that those participants are less computer literate^(54)^. Furthermore, there was an imbalance between the research conditions in the number of participants and covariates. We adjusted our models for sex, educational level and BMI to correct for differences in these covariates between the research conditions. Finally, it is important to note that our study was conducted in times of the COVID-19 pandemic, which could have implications for the stability of our findings over time. Nevertheless, we found that a majority of the participants (82 %) reported to not have changed their food purchases due to COVID-19, suggesting that conditions surrounding the COVID-19 pandemic did not have a major effect on our findings. Poelman *et al.* also demonstrated that most Dutch adults did not change their eating behaviours (83 %) or food purchases (73 %) during the lockdown^(55)^. During the lockdown in the Netherlands, pubs and restaurants were closed while supermarkets and local food shops such as butchers and bakeries remained open. Pubs and restaurants, however, reopened their doors again before the start of our study.

It is unknown whether our findings are generalisable to other countries. Although the effects of health-related food taxes are demonstrated across countries^(11)^, responses to taxes may differ because of different economic and sociocultural contexts. Therefore, repetition of our study in other countries will be of value.

## Conclusions

This study aimed to investigate the effects of an SSB tax and a nutrient profiling tax based on Nutri-Score on SSB purchases and healthy food purchases in a virtual supermarket. We found that the nutrient profiling tax is effective in decreasing SSB purchases as well as in increasing the overall healthiness and decreasing the energy content of the total weekly food shopping basket; in case of an SSB tax, effects were only observed on SSB purchases. These findings implicate that a nutrient profiling tax targeting a wide range of foods and beverages with a low nutritional quality seems to have larger beneficial effects on consumer food purchases than taxation of SSB alone.

## Supplementary material

For supplementary material accompanying this paper visit https://doi.org/10.1017/S1368980021004547. click here to view supplementary material
