# Supplementary material for: The effects of a sugar-sweetened beverage tax and a nutrient profiling tax based on Nutri-Score on consumer food purchases in a virtual supermarket: a randomised controlled trial
Source: Public Health Nutr. 2021 Nov 3;25(4):1105–17. doi: 10.1017/S1368980021004547 (PMC9991614; doi:10.1017/S1368980021004547)
Supplement: Supplementary file 1 [file S1368980021004547sup.zip › S1368980021004547sup001.docx]

SUPPLEMENTARY MATERIAL

**Supplemental Table S1.** Overview of non-alcoholic beverages and the number of taxed products in the experimental conditions in the virtual supermarket.

| Non-alcoholic beverage category | Total products (*n*) | Taxed products in SSB tax and nutrient profiling tax condition (*n*) |  |  |
| --- | --- | --- | --- | --- |
| Fruit juices | 8 | 0 |  |  |
| Fruit and juice drinks | 12 | 10 | Multi-vitamin drinks | - 1.5 L carton (premium brand) - 1.5 L carton (own-brand) - 6x 200 mL carton (premium brand) - 10x 200 mL carton (own-brand) |
|  |  |  | Double fruit juices | - 1.5 L carton (premium brand) - 1.5 L carton (own-brand) |
|  |  |  | Sparkling fruit drinks | - 1.5 L carton (premium brand)^a^ - 1.5 L carton (own-brand)^a^ - 6x 200 mL carton (premium brand)^a^ - 6x 200 mL carton (own-brand)^a^ |
| Soft drinks | 32 | 16 | Cola | - 1.5 L bottle (premium brand) - 1.5 L bottle (own-brand) - 6x 330 mL can (premium brand) - 6x 330 mL can (own-brand) - 500 mL bottle (premium brand) |
|  |  |  | Orange lemonades | - 1.5 L bottle (premium brand) - 1.5 L bottle (own-brand) - 500 mL bottle (premium brand) |
|  |  |  | Lemon drinks | - 1.5 L bottle (premium brand) - 1.5 L bottle (own-brand) |
|  |  |  | Cassis | - 1.25 L bottle (premium brand) - 1.5 L bottle (own-brand) |
|  |  |  | Tonic | - 1 L bottle (premium brand) |
|  |  |  | Iced tea | - 1.5 L bottle (premium brand)^a^ - 1.5 L bottle (own-brand)^a^ - 500 mL bottle (premium brand)^a^ |
| Sports and energy drinks | 8 | 6 | Sports drinks | - 6x 330 mL bottle (premium brand) - 6x 500 mL bottle (own-brand)^a^ - 500 mL bottle (premium brand) |
|  |  |  | Energy drinks | - 4x 250 mL can (premium brand) - 4x 250 mL can (own-brand) - 250 mL can (premium brand) |
| Syrups | 4 | 2 | Lemonade syrups | - 750 mL bottle (premium brand) - 750 mL bottle (own-brand) |
| Waters | 13 | 0 |  |  |
| Coffee and tea^b^ | 7 | 0 |  |  |
| Alcohol replacements | 2 | 0 |  |  |
| Total | 86 | 34 (40%) |  |  |

^a^ Beverages were taxed €0.21 per litre in the SSB tax condition. All other beverages were taxed €0.28 per litre in the SSB tax condition; ^b^ The weight of coffee and tea is for the unprepared product.

**Supplemental Table S2.** Characteristics of participants who completed the shop and those who dropped out.

|  | Total randomized  (*n* 2,744) | | Completed virtual supermarket shop  (*n* 404) | | Dropped out  (*n* 2,340) | |
| --- | --- | --- | --- | --- | --- | --- |
|  | *n* or mean | % or SD | *n* or mean | % or SD | *n* or mean | % or SD |
| Age (years), mean and SD | 55.9 | 16.4 | 48.5 | 15.7 | 57.2 | 16.1 |
| Educational level, *n* and % |  |  |  |  |  |  |
| Low | 951 | 34.7 | 70 | 17.3 | 881 | 37.6 |
| Moderate | 974 | 35.5 | 147 | 36.4 | 827 | 35.3 |
| High | 819 | 29.8 | 187 | 46.3 | 632 | 27.0 |
| Responsibility grocery, *n* and % |  |  |  |  |  |  |
| Largely responsible | 942 | 34.3 | 141 | 34.9 | 801 | 34.2 |
| Totally responsible | 1,802 | 65.7 | 263 | 65.1 | 1,539 | 65.8 |
